# Editorial: Advancing machine perfusion: navigating challenges in abdominal organ transplantation

**DOI:** 10.3389/frtra.2026.2001083

**Published:** 2026-10-06

**Authors:** Aleah L. Brubaker, Gabriel T. Schnickel

**Affiliations:** Division of Hepatobiliary and Transplant Surgery, Department of Surgery, University of California San Diego, La Jolla, CA, USA

**Keywords:** kidney transplantation, liver transplantation, organ donation, organ preservation, perfusion, transplantation

For decades, cold ischemic time dictated the pace of organ transplantation. Static cold storage provided a simple and effective means of slowing ischemic injury, but preservation remained a limiting and largely passive interval between donation and transplantation. Advanced preservation has fundamentally changed that paradigm. Across abdominal transplantation, normothermic regional perfusion (NRP), normothermic machine perfusion (NMP) and hypothermic machine perfusion (HMP) allow clinicians not only to preserve organs, but to resuscitate them, assess their function, modify their biology, and reconsider how and when transplantation occurs.

We developed this Research Topic to explore the questions that emerge as machine perfusion moves from innovation toward broader clinical integration. Early studies appropriately focused on feasibility, safety, and graft outcomes. The questions facing the field are now broader: How can perfusion expand organ utilization? Can an organ be assessed, treated, or even repaired before transplantation? How should these technologies be integrated into increasingly complex donation pathways? And how will they reshape the infrastructure, workforce, and expertise required in organ donation and transplantation? This Research Topic traces the field's evolution from preserving donated organs to improving the organs that are transplanted, along with the people and systems needed to make that possible.

*Advancements in Ex Vivo Machine Perfusion for Abdominal Organ Transplantation* by Olaso et al. lays the groundwork for machine perfusion's role in advanced preservation strategies, outlining opportunities for viability assessment, increased utilization of marginal grafts, and therapeutic intervention. Importantly, its expansion across liver, kidney, pancreas, and intestinal transplantation highlights shared challenges including cost, logistics, standardization, and the need to determine which organs and clinical circumstances derive the greatest benefit.

At the same time, NRP is changing what is possible during organ recovery itself. Assouline et al*.* describe conversion from veno-arterial extracorporeal membrane oxygenation to abdominal NRP following unsuccessful extracorporeal cardiopulmonary resuscitation in a patient with Stanford type A aortic dissection. Despite an extraordinarily complex clinical scenario, abdominal NRP (A-NRP) facilitated successful organ recovery and transplantation and highlighted how innovative preservation techniques can expand donation opportunities. Yet NRP remains the subject of ongoing ethical debate, particularly around the restoration of regional circulation after circulatory death and the safeguards required to prevent cerebral reperfusion. These concerns continue to shape public and professional discourse, and some health systems do not currently permit NRP recovery. As adoption expands, transparency, rigorous safeguards, and continued engagement with clinicians, ethicists, donor families, and the public will remain essential.

Machine perfusion also creates an opportunity to reconsider the purpose of preservation itself. Mayoral et al. review pharmacologic supplementation during hypothermic kidney preservation, identifying a substantial body of preclinical investigation targeting ischemia-reperfusion injury pathways. Although clinical translation remains limited, this work highlights an important conceptual shift that preservation solutions and perfusates can become vehicles for targeted therapy during *ex vivo* organ perfusion.

Tropp et al. explore this concept through liver regeneration. Their review describes the complex cellular and molecular networks governing hepatic regeneration and proposes *ex vivo* machine perfusion as a bridge between mechanistic biology and clinical transplantation. By maintaining whole-organ architecture in a controlled environment, perfusion provides an opportunity for regenerative pathways to be measured, manipulated, and augmented before transplantation. In this framework, the goal moves beyond preserving the organ that was donated toward improving the organ that will ultimately be transplanted.

Technological transformation, however, cannot occur independently of the people and systems responsible for delivering it. Mullens et al. examine machine perfusion from the perspective of the next generation of transplant surgeons. Perfusion may facilitate daytime transplantation and create new opportunities for physiologic assessment, decision-making, and translational research. At the same time, it changes traditional training experiences and introduces new technical and cognitive competencies. The transplant workforce must therefore evolve alongside the technology.

Viewed together, the contributions in this Research Topic begin to answer the broader questions that now confront the field. Perfusion can expand organ utilization by recovering organs that might otherwise be lost and by providing additional information to guide acceptance decisions. It is transforming the organ from something that is simply preserved into something that can be assessed, treated, and potentially repaired before transplantation. At the same time, technologies such as NRP and *ex vivo* perfusion are becoming increasingly interconnected within more complex donation and transplantation pathways, requiring coordination across OPOs, donor hospitals, recovery teams, transplant centers, and perfusion platforms. Their successful integration will therefore depend not only on technological capability, but on the infrastructure, workforce, expertise, ethical frameworks, and systems of care required to deliver them safely and equitably. In this sense, machine perfusion is becoming more than a preservation technology. It is emerging as an enabling platform across the transplantation pathway.

This evolution also raises important questions. Perfusion requires personnel, infrastructure, expertise, and financial resources that are not uniformly available. As its use expands, the field must determine not simply whether these technologies improve individual graft outcomes, but how they affect organ utilization, access to transplantation, cost, sustainability, workforce demands, and equity. Enhanced data collection and multicenter collaboration will be essential to identify which approaches provide meaningful benefit, for which organs and recipients, and under what circumstances.

Machine perfusion began as a strategy for organ preservation. The contributions to this collection suggest that its greater promise may be as a catalyst through which transplantation itself can be reimagined.

